# LGBTQ+-Inclusive Language in Patient-Reported Outcome Measures for Acne Vulgaris

**DOI:** 10.1001/jamadermatol.2024.5077

**Published:** 2024-12-04

**Authors:** Twan Sia, Farah Abou-Taleb, Howa Yeung, Anne Lynn S. Chang

**Affiliations:** 1 Department of Dermatology, Stanford University School of Medicine, Redwood City, California; 2 Department of Dermatology, Emory University School of Medicine, Atlanta, Georgia; 3 Associate Editor, *JAMA Dermatology*

## Abstract

This qualitative study examines whether patient-reported outcomes measures for acne incorporate LGBTQ^+^-inclusive language.

Lesbian, gay, bisexual, transgender, queer/questioning, and other (LGBTQ+) individuals face barriers to health care,^1^ and those with acne have high depression and suicidal ideation risk.^2^ Transmasculine patients receiving gender-affirming testosterone therapy have a high incidence of acne, and optimal treatment regimens need further investigation. Patient-reported outcome measures (PROMs) are developed with importance, feasibility, and useability in mind, and inclusive language affects these qualities. Expert guidelines recommend using PROMs, but whether these instruments incorporate LGBTQ+-inclusive language to appropriately capture outcomes remains unclear.^3^

## Methods

We conducted an inductive thematic analysis of LGBTQ+-inclusive language in 22 PROMs on acne. PROMs were identified by a PubMed search using terms listed in a systematic review^4^ and were included if all language were accessible. Relevant items were those with (1) third-person gendered pronouns/nouns or (2) associations with interpersonal relationships or social situation(s). We defined LGBTQ+-inclusive language per the National Institutes of Health style guide.^5,6^ Three investigators with LGBTQ+ health expertise and/or board certification in dermatology (T.S., F.A., and A.C.) developed a consensus codebook informed by a grounded theory approach that identifies LGBTQ+-inclusive language. Themes were (1) nonassumption of heteronormativity; (2) gender-neutral language; and (3) inclusion of a spectrum of LGBTQ+ identities (eTable in Supplement 1). This study was determined to be institutional review board exempt by Stanford University, as it was not considered human participants research, and informed consent was waived.

## Results

Sixteen PROMs were included: 9 acne-specific (56 relevant items of 177 total items), 4 dermatology-specific (28 of 94 items), and 4 health-related (43 of 206 items) (Table 1). One health-related PROM did not contain any items relevant for analysis. We found LGBTQ+-noninclusive language in 4 of 9 acne-specific PROMS (Acne Disability Index [ADI], Acne Quality of Life Scale [AQOL], Acne-QoL, and Cardiff Acne Disability Index [CADI]); 1992 version), but not in dermatology-specific or health-related PROMs.

**Table 1.** Types of Patient-Reported Outcome Measures (PROMs) Analyzed for LGBTQ+-Inclusive Language

Type of PROM	PROM source (original publication year)	No.		No. of items with LGBTQ+ inclusive language
		Items in PROM	Relevant items^a^	
Acne-specific	Acne Disability Index (1989)	10	6	5
	Assessment of the Psychological and Social Effects of Acne (1991)	15	6	6
	Cardiff Acne Disability Index (1992; adjusted in 2021)	5 (1992 Version)	2 (1992 Version)	1 (1992 Version)
		5 (2021 Version)	2 (2021 Version)	2 (2021 Version)
	Acne-QoL (1996)	19	4	3
	Acne Quality of Life Scale (1998)	9	9	7
	Acne Severity and Impact Scale (2015)	17	2	2
	CompAQ (2018)	20	8	8
	Acne-Q (2019)	63	11	11
	Acne Impact on Adult Daily Life (2021)	14	6	6
Dermatology-specific	Dermatology Life Quality Index (1994)	10	3	3
	Childrens’ Dermatology Life Quality Index (1995)	10	2	2
	Dermatology-Specific Quality of Life (1997)	45	12	12
	Skindex-29 (1997)	29	11	11
Health-related	UK Sickness Profile (1972)	136	37	37
	EuroQol 5-Dimension (1990)	5	0	NA
	36-Item Short Form Survey Instrument (1992)	36	4	4
	Patient-Reported Outcomes Measurement System–Anxiety (2011)	29	2	2

Abbreviations: LGBTQ+, lesbian, gay, bisexual, transgender, queer/questioning, and other; NA, no relevant items identified for analysis.

^a^ Relevant items were defined as those that included (1) third person pronouns or nouns that could be gendered or (2) items assessing an association with interpersonal relationships or social situations. Items were determined to contain LGBTQ+-inclusive language based on the themes in the codebook (see Methods section). PROMs with only summary language and no available license for purchase were excluded. Excluded PROMs were 1 acne-specific (Acne Quality of Life Index), 2 dermatology-specific (Oily Skin Self 9 assessment Scale, Oily Skin Impact Scale), and 1 health-related PROM (Patient Benefit 10 Index). The dermatology-specific and health-related PROMs listed were broader than acne PROMs and did not contain noninclusive language.

Of 6 acne-specific, 3 dermatology-specific, and 1 health-related PROMs that asked about intimate relationships, heteronormative language was identified in 3 of 6 acne-specific PROMs (CADI, ADI, and Acne-QoL). Three acne-specific, 3 dermatology-specific, and 1 health-related PROMs used nonheteronormative terminology, such as *partner*.

Gendered language of *brothers and sisters* rather than the gender-neutral term *sibling* was found in AQOL. All PROMs contained items with nongendered pronouns, such as I or you rather than he or she. Two acne-specific PROMs (Acne-QoL, AQOL) used language inclusive of some but not all LGBTQ+ identities.

## Discussion

Using LGBTQ+-inclusive language may promote the acquisition of accurate and relevant data for patient care and clinical trials and even enhance patient-clinician relationships. Table 2 contains suggestions for LGBTQ+-inclusive language, but content validity and psychometric properties need to be rigorously tested before adoption. Inclusion of diverse populations in development and validation may provide greater understanding of potential language association.

**Table 2.** Principles of LGBTQ+-Inclusive Language Gathered from Inductive Thematic Analysis^a^

NIH guideline section title	Principles of LGBTQ+ inclusive language	Examples of noninclusive language (PROM, item No.)	Suggested revised language (why this language is more inclusive)^b^
Sexual orientation	Nonassumption of heteronormativity	“Do you think that having acne during the last month interfered with… relationships with members of the opposite sex?” (Cardiff Acne Disability Index 1992, item 2)	“…relationships with people you are interested in…” (does not assume heterosexuality)
		“Is the way members of the opposite sex respond to you…” (Acne Disability Index, item 6)	
Sexual and gender diverse	Gender-neutral language, not reinforcing the gender binary	“Difficulties in your relationship with your immediate family (eg, parents, brothers, and sisters)” (Acne Quality of Life Scale, item 5)	“… parents, siblings…” (uses gender-neutral language)
Sexual and gender minority populations	Inclusion of a spectrum of LGBTQ^+^ identities	“In this past WEEK, how much was interacting with the opposite sex (or same sex if gay or lesbian) a problem for you because of your facial acne?” (Acne-QoL, item 14)	“… interacting with people you are interested in…” (this wording is applicable to any sexual orientation or gender identity)
		“Feeling rejected in a romantic relationship because of the effect of acne on your appearance” (Acne Quality of Life Scale, item 8)^1^	“Feeling rejected in an intimate relationship…” or consider moving to “answer only if applicable” section (considers the experience of asexual and/or aromantic individuals)

Abbreviations: LGBTQ+, lesbian, gay, bisexual, transgender, queer/questioning, and other; NIH, National Institutes of Health; QOL, quality of life; PROM, patient-reported outcome measures.

^a^ Examples of LGBTQ^+^ inclusive and noninclusive language in acne-specific PROMs and suggested revised language with reasons for the revised language are shown that are consistent with NIH guides, terms, and definitions.^5^

^b^ The suggested revised language requires vetting by consensus experts and validation and evaluation in a clinical setting by patients or patient advocates.

Modifying PROM language to be more inclusive is challenging. Common stipulations in licensing agreements are to not alter the language, partially to prevent users from inappropriately changing outcomes without revalidating them. Adjusting the language for LGBTQ+ inclusivity might affect comprehension and require revalidation. Because the expectation that these legacy PROMs might be updated (especially since some are owned by the medical industry) may be unrealistic, we direct readers to the PROMs already containing LGBTQ+-inclusive language in Table 1. PROMs developed using item-response/Rasch measurement theory may facilitate future adjustments to language, as it would not change the overall grading of the instrument.

In 2021, the authors of CADI modified its wording from “relationships with members of the opposite sex” to “intimate personal relationships.”^7^ However, to our knowledge, no validation study was performed with the revised wording. Thus, addressing this health care barrier requires acknowledgment of outdated wording, performing in-depth validation research, and discontinuing dissemination of outdated versions. Limitations of our work included analysis of only English-language PROMs.

## Notes

Supplement 1. eTable. Consensus codebook with definitions relevant for LGBTQ+ studies and summary of LGBTQ+ inclusive and non-inclusive language findings

Supplement 2. Data sharing statement
