# Supplementary material for: LGBTQ+-Inclusive Language in Patient-Reported Outcome Measures for Acne Vulgaris
Source: JAMA Dermatol. 2024 Dec 4;161(1):108–10. doi: 10.1001/jamadermatol.2024.5077 (PMC11618628; doi:10.1001/jamadermatol.2024.5077)
Supplement: Supplement 1. — eTable. Consensus codebook with definitions relevant for LGBTQ+ studies and summary of LGBTQ+ inclusive and non-inclusive language findings [file jamadermatol-e245077-s001.pdf]

Supplemental Online Content

Sia T, Abou-Taleb F, Yeung H, Chang ALS. LGBTQ⁺-inclusive language in patient-reported outcome measures for acne vulgaris. *JAMA Dermatol*. Published online December 4, 2024. doi:10.1001/jamadermatol.2024.5077

eTable. Consensus codebook with definitions relevant for LGBTQ+ studies and summary of LGBTQ+ inclusive and non-inclusive language findings

This supplemental material has been provided by the authors to give readers additional information about their work.

© 2024 Sia T et al. *JAMA Dermatology*.

eTable. Consensus codebook with definitions relevant for LGBTQ+ studies and summary of LGBTQ+ inclusive and non-inclusive language findings. Themes and definitions are based on National Institutes of Health (NIH) guides available at https://www.nih.gov/nih-style-guide/sex-gender-sexuality and https://www.edi.nih.gov/people/sep/lgbti/safezone/terminology, accessed July 31, 2024.

Themes	NIH published terms and definitions	Codes of inclusive language findings	Codes of non-inclusive language findings
Non-assumption of heteronormativity	Per NIH guidance, heteronormativity is "[t]he assumption that everyone is heterosexual and that heterosexuality is superior to all other sexualities. This includes the often implicitly held idea that heterosexuality is the norm and that other sexualities are 'different' or 'abnormal.'"	Non-heteronormative language (e.g. partner) when asking about intimate relationships	Heteronormative language when asking about intimate relationships
Gender-neutral language, not reinforcing the gender binary	Per NIH guidance, the gender binary is "[t]he disproven concept that there are only two genders, male and female, and that everyone must be one or the other. Also often misused to assert that gender is biologically determined. This concept also reinforces the idea that men and women are opposites and have different roles in society."	Non-gendered pronouns (e.g. you, I) rather than "he" or "she"	Gendered language (e.g. brother, sister) when there are standard gender-neutral alternatives such as "sibling"
Inclusion of a spectrum of LGBTQ⁺ identities	Per NIH guidance, the plus sign "represents those who are part of the community, but for whom LGBTQ does not accurately capture or reflect their identity." And "The plus sign includes other members of the community, such as asexual, genderfluid, nonbinary, or two-spirit, among others."	Answer choices allows for spectrum of LGBTQ⁺ identities instead of required answer choices that may not apply	Inclusion of some LGBTQ+ identities (e.g. gay, lesbian) but not others (e.g. aromantic, bisexual)

© 2024 Sia T et al. *JAMA Dermatology.*
