# Supplementary material for: LGBTQ+-Inclusive Language in Patient-Reported Outcome Measures for Acne Vulgaris
Source: JAMA Dermatol. 2024 Dec 4;161(1):108–10. doi: 10.1001/jamadermatol.2024.5077 (PMC11618628; doi:10.1001/jamadermatol.2024.5077)
Supplement: Supplement 2. — Data sharing statement [file jamadermatol-e245077-s002.pdf]

Data Sharing Statement

Data
Data available: Yes
Data types: Data (not involving human participants)
How to access data: The data that support the findings of this study are openly available in prior published literature. Derived data supporting the findings of this study are available from the corresponding author on request.
When available: With publication

Supporting Documents
Document types: None

Additional Information
Who can access the data: Anyone requesting the data
Types of analyses: For a specified purpose
Mechanisms of data availability: With investigator support
